# Endogenous CSE/Hydrogen Sulfide System Regulates the Effects of Glucocorticoids and Insulin on Muscle Protein Synthesis

**DOI:** 10.1155/2019/9752698

**Published:** 2019-04-07

**Authors:** Ruxia Wang, Kelin Li, Hui Wang, Hongchao Jiao, Xiaojuan Wang, Jingpeng Zhao, Hai Lin

**Affiliations:** Shandong Provincial Key Laboratory of Animal Biotechnology and Disease Control and Prevention, Shandong Agricultural University, 61 Daizong Street, Taian City, Shandong Province 271018, China

## Abstract

**Aims:**

Insulin and glucocorticoids play crucial roles in skeletal muscle protein turnover. Fast-twitch glycolytic fibres are more susceptible to atrophy than slow-twitch oxidative fibres. Based on accumulating evidence, hydrogen sulfide (H_2_S) is a physiological mediator of this process. The regulatory effect of H_2_S on protein synthesis in fast-twitch fibres was evaluated.

**Results:**

A NaHS (sodium hydrosulfide) injection simultaneously increased the diameter of *M. pectoralis major* (i.e., fast-twitch glycolytic fibres) and activated the mammalian target of the rapamycin (mTOR)/p70S6 kinase (p70S6K) pathway. Dexamethasone (DEX) inhibited protein synthesis, downregulated mTOR and p70S6K phosphorylation, and suppressed the expression of the cystathionine *γ*-lyase (CSE) protein in myoblasts. The precursor of H_2_S, L-cysteine, completely abolished the inhibitory effects of DEX. The CSE inhibitor DL-propargylglycine (PAG) completely abrogated the effects of RU486 on blocking the suppressive effects of DEX. The H_2_S donor NaHS increased the H_2_S concentrations and abrogated the inhibitory effects of DEX on protein synthesis. Insulin increased protein synthesis and upregulated CSE expression. However, PAG abrogated the stimulatory effects of insulin on protein synthesis and the activity of the mTOR/p70S6K pathway.

**Innovation:**

These results demonstrated that CSE/H_2_S regulated protein synthesis in fast-twitch muscle fibres, and glucocorticoids and insulin regulated protein synthesis in an endogenous CSE/H_2_S system-dependent manner.

**Conclusions:**

The results from the present study suggest that the endogenous CSE/H_2_S system regulates fast-twitch glycolytic muscle degeneration and regeneration.

## 1. Introduction

Hydrogen sulfide (H_2_S) has historically been considered a toxic environmental gas [1] but is also a physiological mediator [2–4]. Endogenous sulfides, nitric oxide, and carbon monoxide are gastrotransmitters in the mammalian brain [5–8]. Hydrogen sulfide plays various physiological roles in neuromodulation, vascular tone regulation, cytoprotection, oxygen-sensing capacity, inflammatory regulation, and cell growth [9–11]. H_2_S is also associated with glucose homeostasis [12].

Two enzymes, cystathionine *γ*-lyase (CSE) and cystathionine *β*-synthase (CBS), contribute to the formation of endogenous H_2_S in the cytoplasm. The sulfur-containing amino acids, cysteine and homocysteine, are substrates in reactions that produce H_2_S [13, 14]. 3-Mercaptopyruvate sulfur transferase also produces H_2_S in the vascular endothelium [15], and the sulfur-containing amino acids, methionine, homocysteine, cysteine (CS), and 3-mercaptopyruvate, are the primary sources of endogenous H_2_S [13, 16]. Human skeletal muscles express significant amounts of CBS and CSE [17], suggesting that endogenous H_2_S plays an important role in modulating muscle metabolism. Hydrogen sulfide is endogenously generated in rat skeletal muscle and protects against oxidative stress by acting as an antioxidant [18]. H_2_S prevents ischaemia-reperfusion injury-induced cellular damage in cultured myotubes subjected to sequential hypoxia and normoxia and in vivo (mouse hind limb) models [19]. However, researchers have not clearly determined whether H_2_S exerts antihypertensive, anti-inflammatory, and antioxidant effects on healthy skeletal muscle and on skeletal muscle affected by metabolic syndromes [20].

Cachexia, sarcopenia, and general muscle atrophy resulting from disuse and/or prolonged bed rest are the ultimate consequences of ageing and a variety of acute and chronic illnesses, and these conditions have received more attention in recent decades [21]. The skeletal muscle fibre type profoundly impacts muscle diseases, including certain muscular dystrophies and sarcopenia, and ageing-induced loss of muscle mass and strength [22]. Fast-twitch glycolytic fibres are more susceptible to age-related atrophy than slow-twitch oxidative fibres [23, 24].

Insulin plays a crucial role in skeletal muscle protein turnover and facilitates human skeletal muscle anabolism [25]. Insulin resistance is involved in the development of muscle atrophy [26, 27]. Glucocorticoids regulate protein metabolism in skeletal muscle and exert catabolic effects that oppose the anabolic effects of insulin [28]. Glucocorticoids are associated with muscle-wasting diseases, which affect the overall metabolic state by increasing catabolism, decreasing anabolism, or both [29]. Therefore, we hypothesized that H_2_S would function as a signalling molecule to regulate skeletal muscle protein synthesis.

The mammalian target of the rapamycin (mTOR) signalling pathway is a central mediator of metabolism and growth and acts as a central regulator of protein metabolism [30]. A variety of different stimuli that control protein synthesis and skeletal muscle mass regulate mTOR signalling in skeletal muscle [31, 32]. H_2_S exerts cardioprotective effects by activating the AMPK/mTOR pathway [33]. Therefore, we investigated whether the mTOR pathway is a target of H_2_S in the present study.

The present study used broiler chicks as the experimental model because the breast muscle of the boiler chick primarily comprises fast-twitch muscle fibres. The effect of a NaHS injection on muscle development was evaluated. We measured the inhibitory and stimulatory effects of dexamethasone (DEX), a synthetic glucocorticoid exhibiting a high affinity for glucocorticoid receptors and insulin, respectively, on protein synthesis in myoblasts and investigated the regulatory roles of H_2_S in skeletal muscle protein synthesis and the activation of the mTOR/p70S6 kinase (p70S6K) pathway.

## 2. Results

### 2.1. Intraperitoneal Injection of NaHS Stimulated Breast Muscle Development

**Figure 1:**
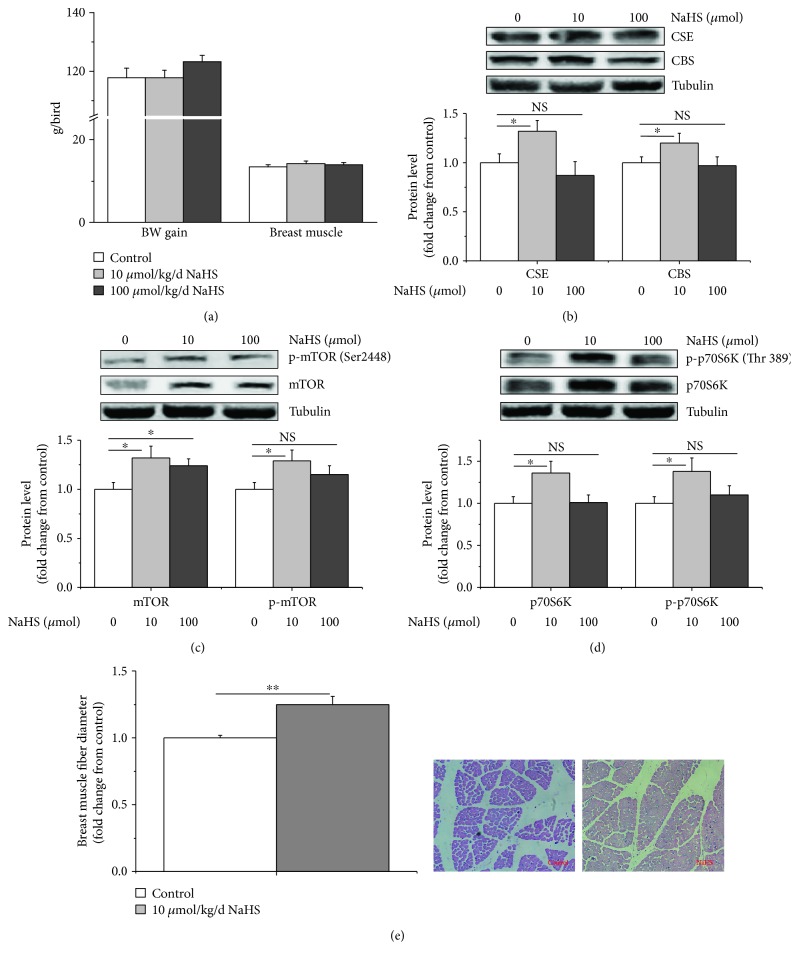
Effects of an intraperitoneal injection of NaHS (10 *μ*mol or 100 *μ*mol/kg BW) on muscle development, CSE expression, and the mTOR/p70S6K signalling pathway. (a) BW gain and breast muscle mass, (b) CSE and CBS protein levels, (c) total mTOR and phospho-mTOR (Ser 2448) (p-mTOR) protein levels, (d) p70S6K or p-p70S6K (Thr 389) protein levels, and (e) results of the morphological analysis of the sections and diameters of *M. pectoralis major* of broilers. The data are presented as the mean ± SEM (*n* = 6); ^∗^ *P* < 0.05; ^∗∗^ *P* < 0.01.

The NaHS injection had no detectable influence (*P* > 0.05) on body weight or breast muscle mass (Figures 1(a) and S2). At a dose of 10 *μ*mol/kg/d, NaHS increased the CSE and CBS protein levels (*P* < 0.05, Figures 1(b) and S2). The NaHS treatment (10 *μ*mol) upregulated the total mTOR, phosphorylated mTOR, and phosphorylated p70S6K protein levels (*P* < 0.05) (Figures 1(c), 1(d), and S2). In contrast, a high-dose NaHS treatment (100 *μ*mol) had no detectable influence on the CBS and CSE protein levels and mTOR and on p70S6K phosphorylation but increased the total mTOR level (*P* < 0.05). We repeated the NaHS treatment (10 *μ*mol/kg body weight (BW)) and observed that the diameters of *M. pectoralis major* fibres were increased (Figures 1(e) and S2).

### 2.2. DEX Inhibited CSE Expression, Protein Synthesis, and the Activity of the mTOR/p70S6K Pathway

**Figure 2:**
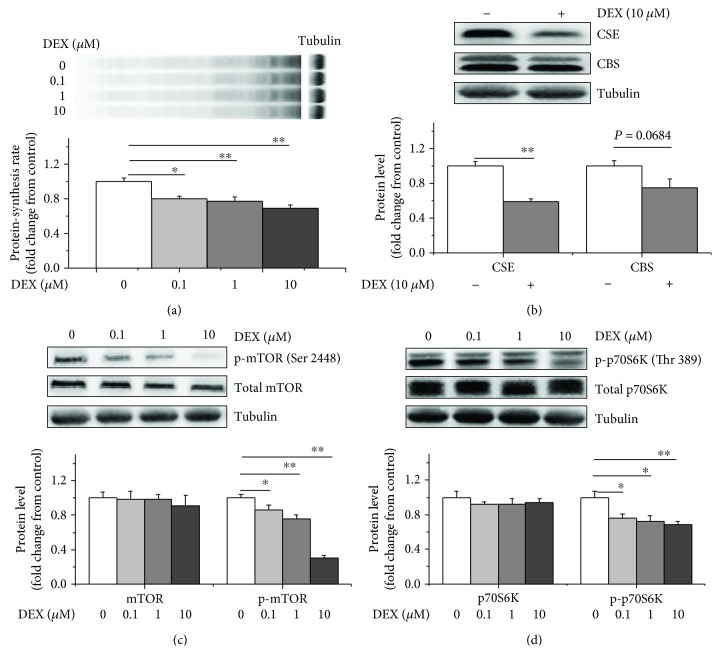
DEX inhibited protein synthesis, CSE expression and the mTOR/p70S6K signalling pathway. Myoblasts were incubated with different doses of DEX (0, 0.1, 1, or 10 *μ*M) for 6 h. All cells were then incubated with puromycin (10 *μ*M) for 30 min to measure the protein synthesis rates. Cell lysates were immunoblotted with specific antibodies. (a) Protein synthesis was measured with an antibody against puromycin, (b) CSE and CBS protein expression levels, (c) total mTOR and p-mTOR (Ser 2448) protein levels, and (d) p70S6K or p-p70S6K (Thr 389) protein levels. The data are presented as the mean ± SEM (*n* = 6); ^∗^ *P* < 0.05; ^∗∗^ *P* < 0.01.

We first investigated whether the H_2_S synthetases, CSE and CBS, participated in the DEX-mediated inhibition of protein synthesis. At all concentrations (0.1, 1, and 10 *μ*mol), the DEX treatment significantly inhibited (*P* < 0.05) protein synthesis compared to the control treatment (Figures 2(a) and S3A). The DEX treatment decreased the CSE and CBS protein levels (*P* < 0.01) compared to the control treatment (*P* = 0.068, Figures 2(b) and S3B). The DEX treatment produced no significant effect (*P* > 0.05) on the total mTOR or p70S6K levels but produced significant decreases (*P* < 0.05) in the phosphorylated mTOR (Ser 2448) and p70S6K (Thr 389) levels compared to the control treatment (Figures 2(c), 2(d), and S3C).

### 2.3. RU486 Reversed the Effects of DEX on Suppressing CSE Expression, Protein Synthesis, and the Activity of the mTOR/p70S6K Pathway

**Figure 3:**
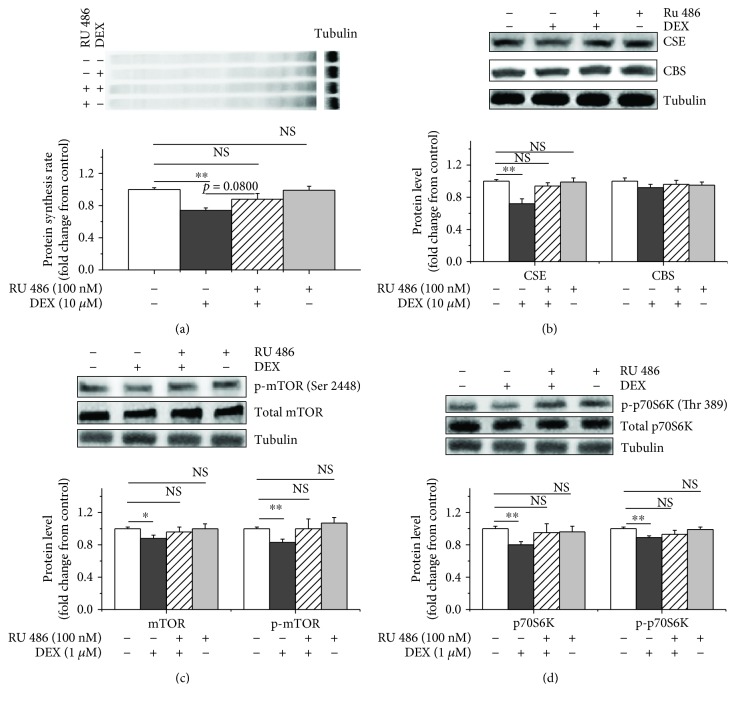
The glucocorticoid receptor mediates the inhibitory effects of DEX on protein synthesis and CSE expression. Myoblasts were preincubated with or without RU 486 (100 nM) for 30 min. Cells were then incubated with or without DEX (10 *μ*M) for 6 h, after which they were incubated with puromycin (10 *μ*M) for 30 min to measure protein synthesis rates. Cell lysates were immunoblotted with specific antibodies. (a) Protein synthesis was measured with an antibody against puromycin, (b) CSE and CBS levels were detected by immunoblotting using CSE and CBS antibodies, (c) antibodies against mTOR or p-mTOR (Ser 2448) were used to detect the protein levels, and (d) antibodies against p70S6K or p-p70S6K (Thr 389) were used to detect the protein. The data are presented as the mean ± SEM (*n* = 6); ^∗^ *P* < 0.05; ^∗∗^ *P* < 0.01.

We treated myoblasts with the glucocorticoid receptor inhibitor RU486 to block the effects of DEX on protein synthesis and confirm that CSE played a role in the inhibitory effects of glucocorticoids on protein synthesis. DEX decreased protein synthesis compared to the control (*P* < 0.01). However, the RU486+DEX treatment attenuated this effect (*P* > 0.05, Figures 3(a) and S4). The DEX treatment significantly decreased the CSE protein levels (*P* < 0.01) compared to the control (Figures 3(b) and S4), but the DEX+RU486 treatment did not alter the CSE protein levels (*P* > 0.05). Neither DEX nor DEX+RU486 treatments altered the CBS protein levels (*P* > 0.05, Figures 3(b) and S4). RU486 attenuated the DEX-induced decreases in the mTOR (*P* < 0.05) and p70S6K (*P* < 0.01) protein levels. RU486 restored the DEX-induced downregulation of mTOR and p70S6K phosphorylation (*P* < 0.01) (Figures 3(c), 3(d), and S4).

### 2.4. L-Cysteine Attenuated the Suppressive Effects of DEX on CSE Expression, Protein Synthesis, and the Activity of the mTOR/p70S6K Pathway

**Figure 4:**
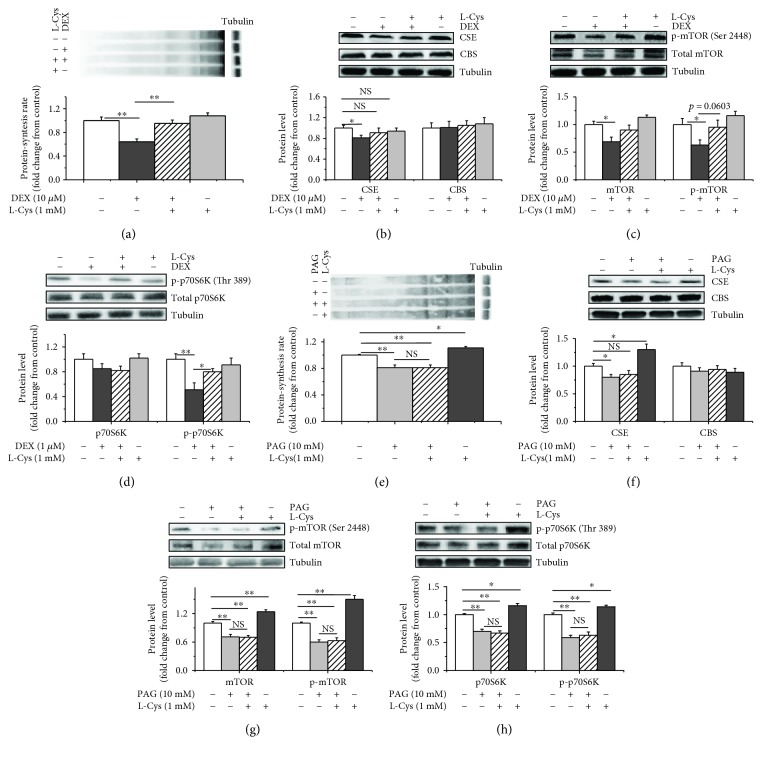
L-cysteine stimulated protein synthesis in chicken myoblasts via H_2_S. Myoblasts were incubated with or without L-cysteine (1 mM) and treated with or without DEX (10 *μ*M) for 6 h (a, b, c, and d). Alternatively, the cells were treated with or without PAG (10 mM) for 6 h (e, f, g, and h). After all treatments were complete, cells were incubated with puromycin (10 *μ*M) for 30 min to evaluate protein synthesis rates. The cell lysates were immunoblotted with specific antibodies. (a, e) Blots were incubated with an antibody against puromycin, (b, f) antibodies against CSE and CBS, (c, g) antibodies against mTOR or p-mTOR (Ser 2448), and (d, h) antibodies against p70S6K or p-p70S6K (Thr 389). The data are presented as the mean ± SEM (*n* = 6); ^∗^ *P* < 0.05; ^∗∗^ *P* < 0.01.

We subsequently evaluated the effects of a substrate of H_2_S synthetase, L-cysteine, on the DEX-mediated inhibition of protein synthesis. L-Cysteine supplementation attenuated the inhibitory effects of DEX on protein synthesis (*P* < 0.05, Figures 4(a) and S5A). DEX decreased the CSE protein levels compared to the control (*P* < 0.01) but had no effect on the CBS protein levels compared to the control (*P* > 0.05, Figures 4(b) and S5A). The DEX+L-cysteine treatment had no effect on the CSE or CBS protein levels compared to the control (*P* > 0.05). L-Cysteine attenuated the DEX-induced decreases in the total (*P* < 0.05) and phosphorylated mTOR (*P* < 0.05) protein levels to be comparable to those of the control group. However, the DEX+L-cysteine treatment had no effect on the total or phosphorylated mTOR protein levels (*P* > 0.05) compared with the control treatment (Figures 4(c) and S5A). The DEX treatment did not affect the total p70S6K levels, but it decreased the phosphorylated p70S6K levels (*P* < 0.05). L-Cysteine significantly (*P* < 0.05) increased the phosphorylated p70S6K levels compared with the control treatment (Figures 4(d) and S5A).

We treated myoblasts with DL-propargylglycine (PAG) and L-cysteine to further evaluate the effects of L-cysteine. The L-cysteine treatment increased the CSE protein levels (*P* < 0.05), and PAG decreased the CSE protein levels (*P* < 0.05) (Figures 4(f) and S5B) compared to the control. No significant (*P* > 0.05) differences in the total protein expression levels were observed between the control and L-cysteine+PAG groups (Figures 4(f) and S5B). The L-cysteine treatment increased the protein synthesis rates (*P* < 0.05) (Figures 4(e) and S5B), total mTOR (*P* < 0.01) (Figure 4(g)) and p70S6K protein levels (*P* < 0.01) (Figures 4(h) and S5B), and phosphorylated mTOR (*P* < 0.01) (Figures 4(g) and S5B) and p70S6K protein levels (*P* < 0.01) (Figures 4(h) and S5B). PAG decreased the protein synthesis rates (*P* < 0.01) (Figure 4(e)), decreased the total mTOR (*P* < 0.05) and p70S6K protein levels (*P* < 0.05) (Figures 4(g) and 4(h)), and suppressed the phosphorylation of the mTOR (*P* < 0.05) and p70S6K proteins (*P* < 0.05) (Figures 4(g) and 4(h)). The PAG+L-cysteine treatment abrogated the effects of the L-cysteine treatment on the protein synthesis rates (*P* < 0.05), mTOR and p70S6K protein levels (*P* < 0.05) (Figures 4(e), 4(g), and 4(h)), and phosphorylated mTOR and p70S6K protein levels (*P* < 0.05) (Figures 4(g) and 4(h)).

### 2.5. NaHS Attenuated the Suppressive Effects of DEX on CSE Expression, Protein Synthesis, and the Activity of the mTOR/p70S6K Pathway

**Figure 5:**
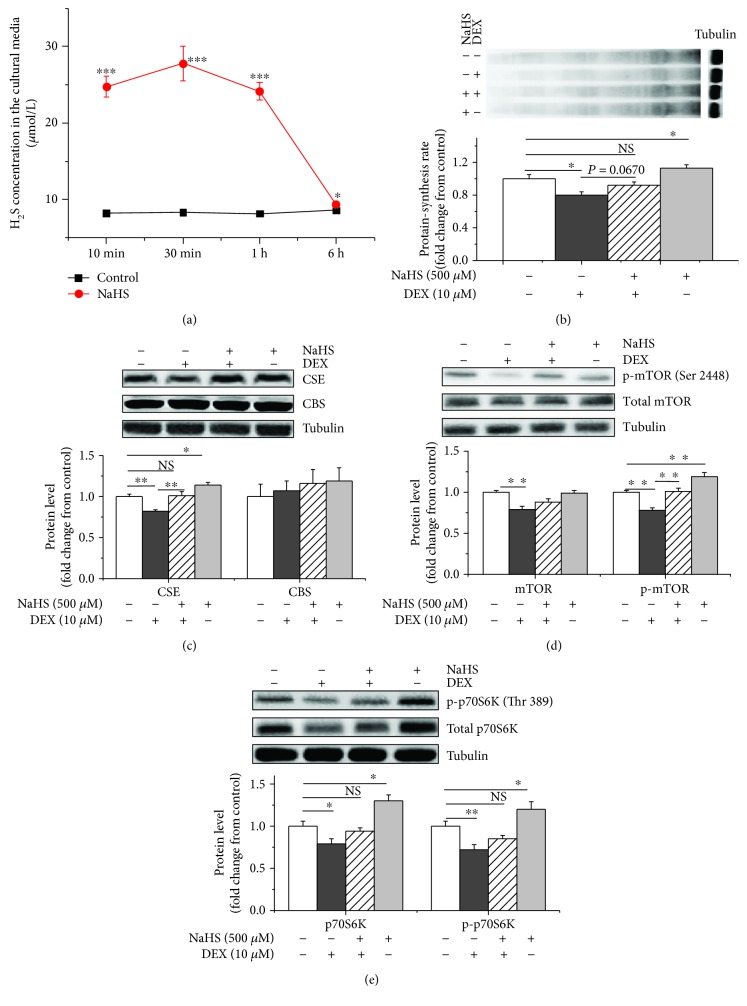
H_2_S stimulated protein synthesis in chicken myoblasts. Myoblasts were incubated with or without NaHS (500 *μ*M). Cells were simultaneously treated with or without DEX (10 *μ*M) for 6 h. (a) The concentration of H_2_S in culture media supplemented with NaHS at different times (10, 30, 60 min, or 6 h). (b) Protein synthesis was measured with an antibody against puromycin. (c) CSE and CBS levels were detected by immunoblotting using CSE and CBS antibodies. (d) The total and phosphorylated mTOR levels were analysed with antibodies against mTOR and p-mTOR (Ser 2448), respectively; (e) the total and phosphorylated p70S6K levels were analysed using antibodies against p70S6K or p-p70S6K (Thr 389), respectively. The data were presented as the mean ± SEM (*n* = 6); ^∗^ *P* < 0.05; ^∗∗^ *P* < 0.01; ^∗∗∗^ *P* < 0.001.

We assessed the effects of NaHS supplementation on myoblast protein synthesis. NaHS supplementation significantly (*P* < 0.05) increased the H_2_S concentration in culture medium within 6 h compared to the control treatment (Figures 5(a) and S6). The NaHS treatment significantly (*P* < 0.05) increased the protein synthesis rates (Figures 5(b) and S6), CSE protein expression levels, and phosphorylated mTOR and p70S6K levels (*P* < 0.05, Figures 5(c)–5(e), and S6) compared to the control treatment. However, the NaHS treatment did not affect the CBS protein expression levels (*P* > 0.05). The NaHS treatment significantly increased the protein synthesis rates and phosphorylated mTOR (Ser 2448) and p70S6K (Thr 389) protein levels compared to the DEX treatment (*P* < 0.05, Figures 5(b), 5(d), and 5(e)).

### 2.6. PAG Reversed the Stimulatory Effects of RU486 on CSE Expression, Protein Synthesis, and the Activity of the mTOR/p70S6K Pathway

**Figure 6:**
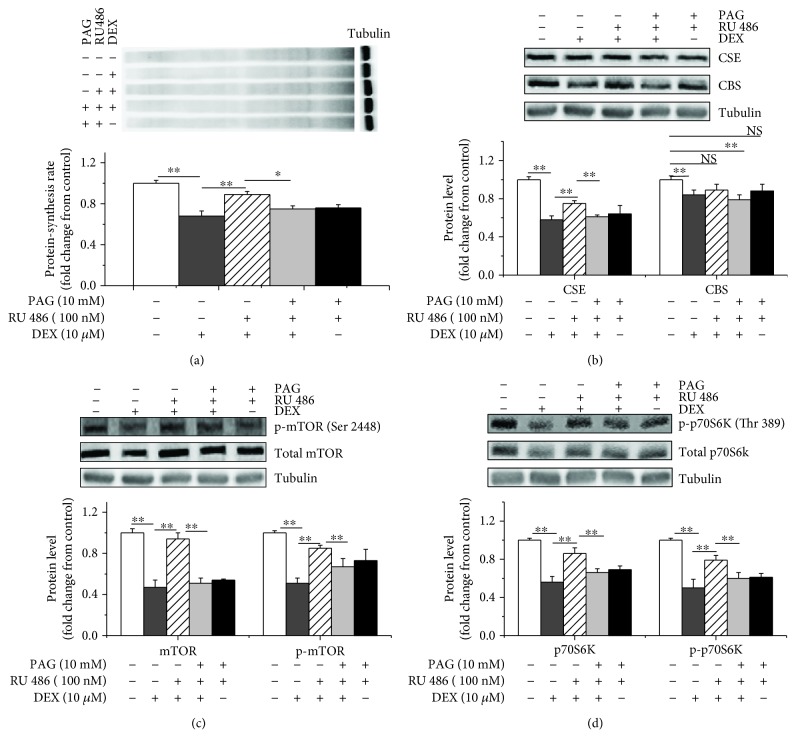
CSE mediated the inhibitory effects of DEX on protein synthesis and the activity of the mTOR/p70S6K signalling pathway. Myoblasts were incubated with DEX (10 *μ*M) for 6 h and treated with or without RU 486 or PAG. All cells were incubated with puromycin (10 *μ*M) for 30 min to evaluate protein synthesis rates. Cell lysates were immunoblotted with the following specific antibodies: (a) puromycin, (b) CSE and CBS, (c) mTOR or p-mTOR (Ser 2448), and (d) p70S6K or p-p70S6K (Thr 389). The data are presented as the mean ± SEM (*n* = 6); ^∗^ *P* < 0.05; ^∗∗^ *P* < 0.01.

The RU486 treatment significantly attenuated the suppressive effects of DEX on the CSE protein expression levels (*P* < 0.05), protein synthesis rates (*P* < 0.05), and mTOR and p70S6K phosphorylation (*P* < 0.05) (Figures 6(a)–6(d), and S7). However, the administration of the PAG treatment in the presence of DEX and RU486 reversed the effects of RU486 and induced significant decreases in CSE expression (*P* < 0.05), protein synthesis rates (*P* < 0.05), and mTOR and p70S6K phosphorylation (*P* < 0.05) (Figures 6(a)–6(d)).

### 2.7. Insulin Stimulated CSE Expression, Protein Synthesis, and p70S6K and mTOR Phosphorylation

**Figure 7:**
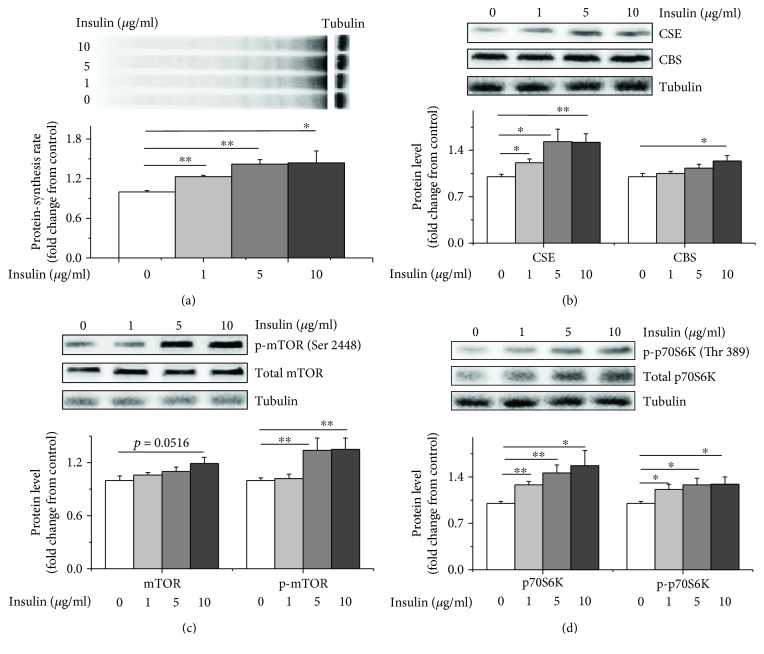
Insulin increased protein synthesis, CSE expression, and the activity of the mTOR/p70S6K signalling pathway. Different doses of insulin (0, 1, 5, or 10 *μ*g/mL) were administered to the myoblasts for 6 h. All cells were then incubated with puromycin (10 *μ*M) for 30 min to evaluate protein synthesis rates. Cell lysates were immunoblotted with the following specific antibodies: (a) puromycin, (b) CSE and CBS, (c) mTOR or p-mTOR (Ser 2448), and (d) p70S6K or p-p70S6K (Thr389). The data are presented as the mean ± SEM (*n* = 6); ^∗^ *P* < 0.05; ^∗∗^ *P* < 0.01.

The insulin treatment (1, 5, or 10 *μ*g/mL) significantly increased the protein synthesis rates (*P* < 0.05, Figures 7(a) and S8) compared to the control treatment. Insulin increased the CSE protein levels (*P* < 0.05) in a dose-dependent manner and increased the CBS protein levels (*P* < 0.05) at its highest dose (10 *μ*g/mL, Figures 7(b) and S8). The insulin treatment also significantly increased mTOR (Ser 2448) and p70S6K (Thr 389) phosphorylation (*P* < 0.05) compared to the control treatment (Figures 7(c), 7(d), and S8).

### 2.8. PAG Abrogated the Stimulatory Effects of Insulin on CSE Expression, Protein Synthesis, and p70S6K and mTOR Phosphorylation

**Figure 8:**
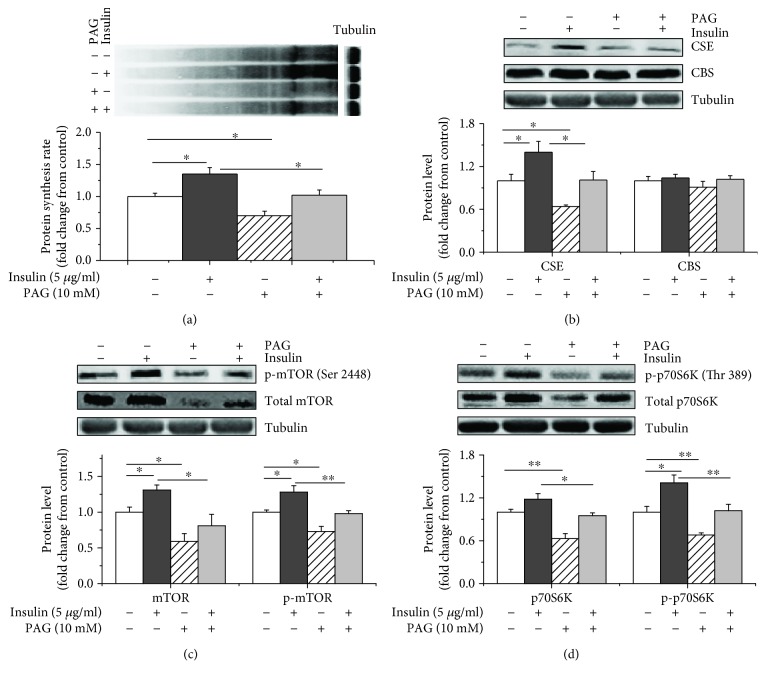
CSE mediated the stimulatory effects of insulin on protein synthesis and the activity of the mTOR/p70S6K signalling pathway. Myoblasts were incubated with insulin (5 *μ*g/mL) for 6 h and treated with or without PAG (10 mM). All cells were then incubated with puromycin (10 *μ*M) for 30 min to evaluate protein synthesis rates. Cell lysates were immunoblotted with the following specific antibodies: (a) puromycin, (b) CSE and CBS, (c) mTOR or p-mTOR (Ser 2448), and (d) p70S6K or p-p70S6K (Thr 389). The data are presented as the mean ± SEM (*n* = 6); ^∗^ *P* < 0.05; ^∗∗^ *P* < 0.01.

We treated cells with PAG in the presence of insulin to determine whether H_2_S was involved in the effects of insulin on myoblast protein synthesis. PAG significantly (*P* < 0.05) suppressed protein synthesis compared to the control group (Figures 8(a) and S9). PAG blocked the insulin-induced increases in protein synthesis rates (*P* < 0.05), and no difference in protein synthesis rates was observed between the PAG-treated and control groups (*P* > 0.05, Figure 8(a)). The insulin treatment also increased the CSE protein levels (*P* < 0.05), and the PAG treatment decreased the CSE protein levels (*P* < 0.05). The PAG+insulin treatment had no effect (*P* > 0.05) on the CSE protein levels compared with the control treatment (Figures 8(b) and S8). However, neither insulin nor PAG significantly affected the CBS protein levels (*P* > 0.05). PAG supplementation significantly (*P* < 0.05) decreased the total and phosphorylated mTOR protein levels (Figures 8(c) and S9), and the insulin treatment increased the total and phosphorylated mTOR protein levels compared to the control treatment (*P* < 0.05). However, the administration of the insulin treatment in the presence of PAG had no effect (*P* > 0.05) on the total and phosphorylated mTOR protein levels compared with the control treatment (Figure 8(c)). The PAG treatment decreased the total and phosphorylated p70S6K protein levels (*P* < 0.05, Figures 8(d) and S9). The insulin treatment increased the phosphorylated p70S6K (p-p70S6K) protein levels (*P* < 0.05) but had no effect on the total p70S6K protein levels (*P* > 0.05). The PAG+insulin treatment did not affect the total p70S6K or p-p70S6K protein levels (*P* > 0.05).

## 3. Discussion

The present study observed a stimulatory effect of NaHS on the development of breast muscle fibres. NaHS increased the protein synthesis rates, activated the mTOR/p70S6K pathway, and increased the CSE protein levels. We investigated the role of H_2_S in the regulatory effects of glucocorticoids and insulin on myocyte protein synthesis. L-Cysteine or NaHS supplementation effectively abolished the inhibitory effects of DEX on protein synthesis, the activity of the mTOR/p70S6K pathway, and CSE protein expression. In contrast, the CSE inhibitor PAG significantly decreased the CSE protein levels and attenuated the stimulatory effects of insulin on protein synthesis and mTOR/p70S6K pathway activity. These results suggest that H_2_S participated in the regulatory effects of insulin and glucocorticoids on protein anabolism in skeletal muscle.

### 3.1. H_2_S Stimulated the Development of Breast Muscle Fibres

NaHS did not alter broiler BW gain or breast muscle mass, but the increased diameter of *M. pectoralis major* indicated a stimulatory effect of NaHS on muscle development. The increased phosphorylated mTOR and p70S6K protein levels in NaHS-treated chicks indicated the activation of the mTOR pathway. Notably, the stimulatory effects of the NaHS treatment were detected at 10 *μ*mol, but not at 100 *μ*mol NaHS, suggesting that NaHS promotes muscle development in a dose-dependent manner. Insulin and glucocorticoids are important hormones that regulate muscle protein metabolism [34]. Therefore, we further investigated the effect of H_2_S on the regulatory effects of insulin and glucocorticoids on protein synthesis.

In this study, increased CBS and CSE protein levels were detected in the 10 *μ*mol NaHS group, suggesting that exogenous H_2_S could upregulate the expression of CBS and CSE. This result was consistent with the study by Wu et al. [35], who reported that the CBS and CSE protein levels in the heart, liver, and kidney tissues of mice were all increased by NaHS supplementation (10-100 *μ*mol/kg/day). Similarly, the NaHS treatment stimulates CBS and CSE expression in the myocardium [36] and kidney [37]. In the present study, the stimulatory effect was not observed in the group supplemented with a high dose of NaHS (100 *μ*mol/kg/day). The effective dose of NaHS showed a tissue-specific pattern, which may be related to the diverse distribution and abundant expression of H_2_S-producing enzymes in the tissues [35]. The underlying mechanism requires further investigation.

### 3.2. H_2_S Abolished the Inhibitory Effects of DEX on Protein Synthesis

The inhibitory effects of glucocorticoids on muscle protein synthesis are well studied [38, 39]. The present study demonstrated that DEX suppressed protein synthesis. mTOR is a central regulator of protein synthesis that plays a role in regulating numerous components, including initiation and elongation factors [40], and mTOR pathway blockade is involved in the inhibitory effects of glucocorticoids on protein synthesis in C2C12 cells [41] and chicken myoblasts [42]. The observation that DEX decreased mTOR and p70S6K phosphorylation indicated that DEX suppressed the activation of the mTOR/p70S6K pathway and suggests that the mTOR/p70S6K pathway is involved in the mechanism regulating the effects of glucocorticoids on protein metabolism. We investigated the effects of glucocorticoids on protein synthesis further via a blockade of the glucocorticoid receptor with RU486. RU486 attenuated the inhibitory effects of DEX on protein synthesis and the expression of the mTOR and p70S6K proteins, suggesting that DEX inhibits protein synthesis via the glucocorticoid receptor [43].

CBS and CSE endogenously synthesize H_2_S from L-cysteine [14, 44, 45], and we measured the CSE and CBS protein levels in this study. Notably, the DEX treatment decreased the CSE protein expression levels, and RU486 partially restored CSE expression (*P* = 0.065). Therefore, endogenous H_2_S production is involved in the effects of DEX treatment on protein synthesis. However, the DEX treatment also decreased the CBS protein expression levels (*P* = 0.068), but RU486 did not significantly affect the CBS levels. This finding suggests that CBS is not the primary enzyme responsible for regulating the effects of DEX on protein synthesis. However, a previous study demonstrated that DEX-treated rats exhibit marked reductions in CBS and CSE expression in the homogenates of mesenteric arterial beds and carotid arteries [40, 46], suggesting a tissue-specific expression pattern for CBS and CSE [13].

We administered a substrate involved in endogenous H_2_S synthesis, L-cysteine, to myoblasts in the presence of DEX. L-Cysteine supplementation significantly increased the CSE protein levels, protein synthesis rates, and mTOR and p70S6K phosphorylation, indicating that L-cysteine attenuated the DEX-induced decrease in protein synthesis rates and the activity of the mTOR/p70S6K pathway. CS may function as a potent biological antioxidant by serving as a source of thiol to regulate intracellular glutathione levels [47]. CS suppresses oxidative stress-induced protein modifications, which decreases protease activity levels and ultimately decreases myofibrillar proteolysis in chick myotubes [48]. We treated cells with the CSE inhibitor PAG in combination with L-cysteine to verify the effects of L-cysteine on myoblast protein synthesis. PAG supplementation in the presence of L-cysteine abrogated the effects of L-cysteine on protein synthesis rates, CSE protein expression, and the activity of the mTOR/p70S6K pathway. However, the combined L-cysteine and PAG treatment did not affect the CBS protein expression levels, indicating that CBS was not responsible for the effects of L-cysteine on protein synthesis. Thus, the stimulatory effects of L-cysteine on protein synthesis and the activity of the mTOR/p70S6K pathway depend on the CSE/H_2_S system.

We investigated whether H_2_S was associated with the DEX-mediated inhibition of protein synthesis. NaHS, a hydrogen sulfide or exogenous H_2_S donor, is used to regulate cardiovascular circulation [49, 50]. NaHS supplementation significantly increased H_2_S concentrations in the culture medium. The NaHS treatment significantly increased the CSE protein expression levels, protein synthesis rates, and mTOR and p70S6K phosphorylation, indicating that H_2_S enhanced protein synthesis in myoblasts. These results are consistent with a study in renal epithelial cells, in which H_2_S stimulated protein synthesis [51]. The NaHS treatment partially attenuated the inhibitory effects of DEX on CSE expression, protein synthesis, and mTOR and p70S6K phosphorylation, suggesting that H_2_S is at least partially responsible for the inhibitory effects of DEX on protein synthesis.

We treated myoblasts with PAG in the presence of DEX and RU486 to confirm the role of H_2_S in the DEX-mediated inhibition of protein synthesis. The PAG treatment significantly suppressed CSE expression, protein synthesis, and the activity of the mTOR/p70S6K pathway compared to the control treatment. These results are consistent with the study by Lee et al., who reported that PAG abrogated the stimulatory effects of tadalafil on protein synthesis and mTOR complex 1 activity in podocytes [52]. PAG also reversed the stimulatory effects of RU486 on protein synthesis, mTOR and p70S6K phosphorylation, and CSE expression. Based on these results, the regulatory effects of glucocorticoids on protein synthesis are at least partially dependent on the endogenous CSE/H_2_S system.

### 3.3. PAG Abrogated the Stimulatory Effects of Insulin on Protein Synthesis

Insulin is a key factor in the mechanism regulating skeletal muscle protein anabolism [53–55]. Insulin successfully increased protein synthesis, the phosphorylated mTOR and p70S6K protein levels, and the expression of the CSE protein in the present study. The activation of intracellular kinases, such as PI3K and mTOR, mediates insulin signalling and affects the phosphorylation of some major effectors involved in the mechanism regulating translation initiation [56]. Insulin increased protein synthesis rates in a dose-dependent manner (0, 1, 5, and 10 *μ*g/mL) and increased mTOR and p70S6K phosphorylation at doses of 5 and 1 *μ*g/mL, respectively, suggesting that the mTOR/p70S6K pathway participates in insulin-induced myoblast protein synthesis. The endogenous CSE/H_2_S system plays an important role in regulating glucose utilization and insulin resistance in 3T3-L1 adipocytes [57, 58] and hepatocytes [59]. The insulin treatment increased the CSE and CBS protein levels in the present study, suggesting that endogenous H_2_S participates in insulin-induced protein synthesis.

We treated myoblasts with PAG in the presence of insulin to confirm the abovementioned hypothesis. PAG abrogated the insulin-induced increase in CSE protein expression to levels comparable to those in the control group, suggesting that PAG suppresses the stimulatory effects of insulin on CSE expression. PAG abolished the stimulatory effects of insulin on protein synthesis and mTOR and p70S6K phosphorylation. Thus, insulin stimulates myoblast protein anabolism in an endogenous CSE/H_2_S-dependent manner.

*S*-Sulfhydration is proposed to mediate most of the effects from H_2_S by producing a hydropersulfide moiety (–SSH) in the CS residues of targeted proteins [60]. Recently, H_2_S was shown to S-sulfhydrate MEK1, the upstream activator of ERK1/2, at CS 341 and induce ERK1/2 phosphorylation, which subsequently translocates to the nucleus and activates PARP-1 activation, in turn improving DNA damage repair and cellular senescence [61]. Therefore, the role of the S-sulfhydration activity of H_2_S in the activation of mTOR requires further investigation.

In conclusion, NaHS stimulated the development of breast muscle. The endogenous CSE/H_2_S system regulated the glucocorticoid-mediated inhibition of protein synthesis and insulin-induced protein anabolism in myoblasts by activating the mTOR/p70SK signalling pathway. The present results highlight the endogenous CSE/H_2_S system as a potential regulator of muscle degeneration and regeneration.

## 4. Innovation

The results demonstrated that the endogenous CSE/H_2_S system participated in the mechanisms regulating protein synthesis in fast-twitch skeletal muscle fibres and glucocorticoid- and insulin-regulated protein synthesis.

## 5. Materials and Methods

### 5.1. Animal Experiment

Day-old male broilers (Arbor Acres) were obtained from a local breeding farm (Shandong, CN), randomly divided into three groups of six chicks and subjected to one of the following treatments: intraperitoneal injections of NaHS at a dose of 10 *μ*mol/kg BW or 100 *μ*mol/kg BW or sham injections of saline (control) twice a day. The initial and final BWs of chicks were recorded, and BW gain was calculated. All experimental chicks were sacrificed at the end of the experiment (8 days old) via exsanguination. The breast muscle was harvested and weighed, and left *M. pectoralis major* (fast-twitch glycolytic fibre type muscle) samples were obtained and snap-frozen in liquid nitrogen for protein analyses.

Animal experiments were repeated to evaluate the development of breast muscle. One-day-old male broilers were randomly divided into two groups of six chicks and subjected to one of the following treatments: intraperitoneal injections of NaHS (10 *μ*mol/kg BW) or sham injections of saline (control) twice a day. All experimental chicks were sacrificed at the age of 8 days via exsanguination. The breast muscle was harvested and weighed, and muscle samples were excised and fixed with 4% paraformaldehyde for morphological analyses.

The Shandong Agricultural University approved this study, which was performed in accordance with the “Guidelines for Experimental Animals” of the Ministry of Science and Technology (Beijing, China).

### 5.2. Cell Culture

SPF chicken eggs were obtained (Jinan SAIS Poultry Co., LTD.) and hatched in an incubator (Haijiang, Beijing, CN). At an embryo age of 15 days, the eggs were placed in the biosafety cabinet (1200, Heal Force) after the eggshell was sterilized with 75% alcohol. Chicken embryos were removed from eggs and placed in glass containers. Breast muscle tissues (*M. pectoralis major*) were separated from the chicken embryo, plated, and cultured in high-glucose Dulbecco's modified Eagle's medium (DMEM; HyClone, Thermo Fisher, Shanghai, CN) supplemented with 10% foetal bovine serum and 1% penicillin/streptomycin (Solarbio, Beijing, CN) in a humidified 5% CO_2_ atmosphere at 37°C until the cells reached approximately 95% confluence. Cells were subjected to the treatments specified in the protocols for the experiments described below.

### 5.3. Treatments

#### 5.3.1. DEX, RU486, L-Cysteine, and NaHS Treatments

Myoblasts were incubated in media supplemented with different concentrations of DEX (0.1, 1, or 10 *μ*M; Shandong, CN) for 6 h to determine the optimal dose (10 *μ*M). L-Cysteine (1 mM; Sigma, Missouri, US) was added to the culture media in the presence of DEX (10 *μ*M), and myoblasts were incubated for 6 h. We treated the myoblasts with NaHS (500 *μ*M, an H_2_S donor; Sigma, Missouri, US) and DEX (10 *μ*M) for 6 h to confirm that H_2_S stimulated protein synthesis. The optimal treatment doses and durations were selected based on the results of previous studies [42, 62, 63]. Cells were treated with a glucocorticoid receptor inhibitor, RU486, to suppress the effects of DEX [64] and confirm that DEX inhibited protein synthesis. We also treated the cells with the CSE inhibitor PAG (10 mM; Sigma, Missouri, US) to inhibit H_2_S formation. The NaHS and PAG concentrations were based on previous trials that used a different gradient. We pretreated the myoblasts with RU486 (100 nM; Sigma, St. Louis, MO, US) and PAG (10 mM) for 1 h prior to the addition of DEX (10 *μ*M).

#### 5.3.2. Insulin and PAG Treatments

Myoblasts were treated with different insulin concentrations (1, 5, or 10 *μ*g/mL; Aladdin, Shanghai, CN) for 6 h to determine the optimal dose (5 *μ*g/mL), i.e., the dose of insulin that would most effectively stimulate protein anabolism [65, 66]. PAG (10 mM) was added to the culture media in the presence of insulin (5 *μ*g/mL) and incubated for 6 h.

#### 5.3.3. Morphological Examination of Breast Muscle

Paraffin-embedded breast muscle tissue was cut into 4 *μ*m thick frontal sections and stained with haematoxylin and eosin (H.E. staining). Sections were examined under a microscope (10 × 20, Olympus, Tokyo, Japan), and muscle fibre diameters were measured using Image-Pro Plus software (Media Cybernetics, Maryland, USA). Approximately 200 muscle fibres were identified and counted in each section to calculate an average muscle fibre diameter for each section.

#### 5.3.4. Protein Synthesis Rate Measurements

Protein synthesis rates were measured using a nonradioactive method [67]. Puromycin (10 *μ*M; Solarbio, Beijing, CN) was added to cell culture media for 30 min after the addition of the treatments listed above, and total proteins were extracted and used to measure protein synthesis rates. Newly synthesized polypeptides were labelled with puromycin at low concentrations to reflect the rate of protein synthesis [67, 68]. The protein-antibody complexes were detected with ECL Plus A and B (Beyotime, Nanjing, Jiangsu, CN), and the results were quantified using the Fusion FX software (Vilber Lourmat, Paris, FR).

#### 5.3.5. H_2_S Concentration Assays

Myoblasts were treated with NaHS (500 *μ*M), and the culture media were collected at 10, 30, 60 min, and 6 h posttreatment. H_2_S concentrations in the media were assessed using a commercial kit (Comin, Jiangsu, CN) and a microplate reader (JET, Guangzhou, CN). In the presence of H_2_S, zinc acetate is reduced to zinc sulfide, and N,N-dimethyl-p-phenylenediamine mono hypochloride (DPMH) subsequently produces methylene blue by the catalysis of ferric chloride. The absorption of methylene blue was detected at 665 nm using a UV-2450 spectrophotometer.

#### 5.3.6. Protein Preparation and Western Blot Analysis

Cells were washed with PBS and lysed in lysis buffer. Supernatants were obtained and used for immunoblotting analyses. Protein concentrations were determined using a BCA protein assay kit (Beyotime, Jiangsu, CN). Tissue samples were homogenized in 1 mL of lysis buffer (Beyotime, Jiangsu, CN) and centrifuged at 12000 g for 10 min at 4°C. The supernatant was collected, and proteins were quantified using a BCA protein assay kit (Beyotime, Jiangsu, CN) according to the manufacturer's protocol.

Eighteen-microgram aliquots of protein were electrophoresed on a 7.5% SDS polyacrylamide gel, and separated proteins were transferred to a polyvinylidene fluoride membrane in Western transfer buffer. The membrane was blocked prior to incubation with the following primary antibodies: anti-phospho-p70S6K (Thr 389), anti-p70S6K, anti-phospho-mTOR (Ser 2448), anti-mTOR (Cell Signaling Technologies, Boston, MA, US), anti-mouse puromycin (Kerafast, Boston, MA, US), anti-CBS (Abcam, Cambridge, UK), anti-CSE (Abcam, Cambridge, UK), and antitubulin (Beyotime, Nantong, Jiangsu, CN). Membranes were washed, and the proteins were probed using horseradish peroxidase-linked anti-rabbit or anti-mouse secondary antibodies. Membranes were exposed to enhanced chemiluminescence plus Western blot detection reagents (Beyotime, Jiangsu, CN). The films were scanned, and the intensities of specific bands were quantified using ImageJ 1.43 software (National Institutes of Health, Bethesda, MD, US). Bands were normalized to tubulin levels in the same sample. Protein molecular weight markers were used to calculate the molecular weights of the proteins in each sample (Figure S9).

### 5.4. Statistical Analysis

The data are presented as the mean ± SEM. The results were analysed using one-way ANOVA and the Statistical Analysis Systems statistical software package (Version 8e; SAS Institute Inc., Cary, NC, US). Differences between the means were evaluated using Duncan's significant difference tests. The means were considered significant at *P* < 0.05 and were considered to be approaching significance at *P* < 0.10.

## Data Availability

The data used to support the findings of this study are available from the corresponding author upon request.

## Abbreviations

BW: Body weight
CBS: Cystathionine *β*-synthase
CSE: Cystathionine *γ*-lyase
DEX: Dexamethasone
H_2_S: Hydrogen sulfide
L-cys: L-Cysteine
mTOR: Mammalian target of rapamycin
NaHS: Sodium hydrosulfide hydrate
PAG: DL-propargylglycine
p70S6K: p70S6 kinase
p-mTOR: Phosphorylated mTOR (Ser 2448)
p-p70S6K: Phosphorylated p70S6K (Thr 389)
RU486: Mifepristone.

## Supplementary Materials

Supplementary Materials The relative expression of cystathionine *γ*-lyase (CSE), cystathionine *β*-synthase (CBS), and 3-mercaptopyruvate sulfur transferase (3-MST) in myoblasts of chicken ^∗^ *P* < 0.05.

## References

[1] Gregorakos L., Dimopoulos G., Liberi S., Antipas G. (1995). Hydrogen sulfide poisoning: management and complications. *Angiology*.

[2] Ali M. Y., Ping C. Y., Mok Y.-Y. P. (2006). Regulation of vascular nitric oxide in vitro and in vivo; a new role for endogenous hydrogen sulphide?. *British Journal of Pharmacology*.

[3] Dong D. L., Zhang Y., Lin D. H. (2007). Carbon monoxide stimulates the Ca2^+^-activated big conductance k channels in cultured human endothelial cells. *Hypertension*.

[4] Stein A., Bailey S. M. (2013). Redox biology of hydrogen sulfide: implications for physiology, pathophysiology, and pharmacology. *Redox Biology*.

[5] Goodwin L. R., Francom D., Dieken F. P. (1989). Determination of sulfide in brain tissue by gas dialysis/Ion chromatography: postmortem studies and two case reports. *Journal of Analytical Toxicology*.

[6] Łowicka E., Bełtowski J. (2007). Hydrogen sulfide (H_2_S)-the third gas of interest for pharmacologists. *Pharmacological Reports*.

[7] Savage J. C., Gould D. H. (1990). Determination of sulfide in brain tissue and rumen fluid by ion-interaction reversed-phase high-performance liquid chromatography. *Journal of Chromatography*.

[8] Warenycia M. W., Goodwin L. R., Benishin C. G. (1989). Acute hydrogen sulfide poisoning. Demonstration of selective uptake of sulfide by the brainstem by measurement of brain sulfide levels. *Biochemical Pharmacology*.

[9] Kimura H. (2017). Hydrogen sulfide and polysulfide signaling. *Antioxidants & Redox Signaling*.

[10] Wang R. (2002). Two’s company, three’s a crowd: can H_2_S be the third endogenous gaseous transmitter?. *The FASEB Journal*.

[11] Yuan S., Pardue S., Shen X., Alexander J. S., Orr A. W., Kevil C. G. (2016). Hydrogen sulfide metabolism regulates endothelial solute barrier function. *Redox Biology*.

[12] Untereiner A., Wu L. (2018). Hydrogen sulfide and glucose homeostasis: a tale of sweet and the stink. *Antioxidants & Redox Signaling*.

[13] Kamoun P. (2004). Endogenous production of hydrogen sulfide in mammals. *Amino Acids*.

[14] Szabó C. (2007). Hydrogen sulphide and its therapeutic potential. *Nature Reviews Drug Discovery*.

[15] Kimura H., Shibuya N., Mikami Y., Kimura Y., Nagahara N. (2010). Vascular endothelium expresses 3-mercaptopyruvate sulfurtransferase and produces H_2_S. *Neuroscience Research*.

[16] Mitidieri E., Tramontano T., Gurgone D. (2018). Mercaptopyruvate acts as endogenous vasodilator independently of 3-mercaptopyruvate sulfurtransferase activity. *Nitric Oxide*.

[17] Chen N. C., Yang F., Capecci L. M. (2010). Regulation of homocysteine metabolism and methylation in human and mouse tissues. *The FASEB Journal*.

[18] Du J. T., Li W., Yang J. Y., Tang C. S., Li Q., Jin H. F. (2013). Hydrogen sulfide is endogenously generated in rat skeletal muscle and exerts a protective effect against oxidative stress. *Chinese Medical Journal*.

[19] Henderson P. W., Jimenez N., Ruffino J. (2011). Therapeutic delivery of hydrogen sulfide for salvage of ischemic skeletal muscle after the onset of critical ischemia. *Journal of Vascular Surgery*.

[20] Veeranki S., Tyagi S. C. (2015). Role of hydrogen sulfide in skeletal muscle biology and metabolism. *Nitric Oxide*.

[21] Palus S., von Haehling S., Springer J. (2014). Muscle wasting: an overview of recent developments in basic research. *International Journal of Cardiology*.

[22] Talbot J., Maves L. (2016). Skeletal muscle fiber type: using insights from muscle developmental biology to dissect targets for susceptibility and resistance to muscle disease. *Wiley Interdisciplinary Reviews: Developmental Biology*.

[23] Braga A. S., Padilha F. G. F., Ferreira A. M. R. (2016). Evaluation of muscle fiber types in German shepherd dogs of different ages. *Anatomical Record*.

[24] Larsson L., Biral D., Campione M., Schiaffino S. (1993). An age-related type IIB to IIX myosin heavy chain switching in rat skeletal muscle. *Acta Physiologica Scandinavica*.

[25] Abdulla H., Smith K., Atherton P. J., Idris I. (2016). Role of insulin in the regulation of human skeletal muscle protein synthesis and breakdown: a systematic review and meta-analysis. *Diabetologia*.

[26] Lee C. G., Boyko E. J., Strotmeyer E. S. (2011). Association between insulin resistance and lean mass loss and fat mass gain in older men without diabetes mellitus. *Journal of the American Geriatrics Society*.

[27] Rudrappa S. S., Wilkinson D. J., Greenhaff P. L., Smith K., Idris I., Atherton P. J. (2016). Human skeletal muscle disuse atrophy: effects on muscle protein synthesis, breakdown, and insulin resistance-a qualitative review. *Frontiers in Physiology*.

[28] Bodine S. C., Furlow J. D. (2015). Glucocorticoids and skeletal muscle. *Advances in Experimental Medicine and Biology*.

[29] Hall D. T., Ma J. F., di Marco S., Gallouzi I. E. (2011). Inducible nitric oxide synthase (iNOS) in muscle wasting syndrome, sarcopenia, and cachexia. *Aging*.

[30] Dennis P. B., Fumagalli S., Thomas G. (1999). Target of rapamycin (TOR): balancing the opposing forces of protein synthesis and degradation. *Current Opinion in Genetics & Development*.

[31] Bolster D. R., Jefferson L. S., Kimball S. R. (2004). Regulation of protein synthesis associated with skeletal muscle hypertrophy by insulin-, amino acid- and exercise-induced signalling. *The Proceedings of the Nutrition Society*.

[32] Goodman C. A., Mayhew D. L., Hornberger T. A. (2011). Recent progress toward understanding the molecular mechanisms that regulate skeletal muscle mass. *Cellular Signalling*.

[33] Yang F., Zhang L., Gao Z. (2017). Exogenous H_2_S protects against diabetic cardiomyopathy by activating autophagy via the AMPK/mTOR pathway. *Cellular Physiology and Biochemistry*.

[34] Rooyackers O. E., Nair K. S. (1997). Hormonal regulation of human muscle protein metabolism. *Annual Review of Nutrition*.

[35] Wu W., Hou C. L., Mu X. P. (2017). H_2_S donor NaHS changes the production of endogenous H_2_S and NO in D-galactose-induced accelerated ageing. *Oxidative Medicine and Cellular Longevity*.

[36] Li N., Wang M. J., Jin S. (2016). The H_2_S donor NaHS changes the expression pattern of H_2_S-producing enzymes after myocardial infarction. *Oxidative Medicine and Cellular Longevity*.

[37] Hou C. L., Wang M. J., Sun C. (2016). Protective effects of hydrogen sulfide in the ageing kidney. *Oxidative Medicine and Cellular Longevity*.

[38] Schakman O., Kalista S., Barbé C., Loumaye A., Thissen J. P. (2013). Glucocorticoid- induced skeletal muscle atrophy. *The International Journal of Biochemistry & Cell Biology*.

[39] Wang R., Jiao H., Zhao J., Wang X., Lin H. (2016). Glucocorticoids enhance muscle proteolysis through a myostatin-dependent pathway at the early stage. *PLoS One*.

[40] Thoreen C. C., Chantranupong L., Keys H. R., Wang T., Gray N. S., Sabatini D. M. (2012). A unifying model for mTORC1-mediated regulation of mRNA translation. *Nature*.

[41] Morgan S. A., Hassan-Smith Z. K., Doig C. L., Sherlock M., Stewart P. M., Lavery G. G. (2016). Glucocorticoids and 11*β*-HSD1 are major regulators of intramyocellular protein metabolism. *The Journal of Endocrinology*.

[42] Wang X., Jia Q., Xiao J., Jiao H., Lin H. (2015). Glucocorticoids retard skeletal muscle development and myoblast protein synthesis through a mechanistic target of rapamycin (mTOR)-signaling pathway in broilers (*Gallus gallus domesticus*). *Stress*.

[43] Kuo T., Harris C. A., Wang J. C. (2013). Metabolic functions of glucocorticoid receptor in skeletal muscle. *Molecular and Cellular Endocrinology*.

[44] Meister A., Fraser P. E., Tice S. V. (1954). Enzymatic desulfuration of *β*-mercaptopyruvate to pyruvate. *Journal of Biological Chemistry*.

[45] Navarra P., Dello Russo C., Mancuso C., Preziosi P., Grossman A. (2000). Gaseous neuromodulators in the control of neuroendocrine stress axis. *Annals of the New York Academy of Sciences*.

[46] d'Emmanuele di Villa Bianca R., Mitidieri E., Donnarumma E. (2015). Hydrogen sulfide is involved in dexamethasone-induced hypertension in rat. *Nitric Oxide*.

[47] Ishii T., Sugita Y., Bannai S. (1987). Regulation of glutathione levels in mouse spleen lymphocytes by transport of cysteine. *Journal of Cellular Physiology*.

[48] Nakashima K., Masaki S., Yamazaki M., Abe H. (2004). Cysteine suppresses oxidative stress-induced myofibrillar proteolysis in chick myotubes. *Bioscience, Biotechnology, and Biochemistry*.

[49] Tian X. Y., Wong W. T., Sayed N. (2012). NaHS relaxes rat cerebral artery in vitro via inhibition of l-type voltage-sensitive Ca^2+^ channel. *Pharmacological Research*.

[50] Wang M. J., Cai W. J., Li N., Ding Y. J., Chen Y., Zhu Y. C. (2010). The hydrogen sulfide donor NaHS promotes angiogenesis in a rat model of hind limb ischemia. *Antioxidants & Redox Signaling*.

[51] Lee H. J., Mariappan M. M., Feliers D. (2012). Hydrogen sulfide inhibits high glucose-induced matrix protein synthesis by activating AMP-activated protein kinase in renal epithelial cells. *Journal of Biological Chemistry*.

[52] Lee H. J., Feliers D., Mariappan M. M. (2015). Tadalafil integrates nitric oxide-hydrogen sulfide signaling to inhibit high glucose-induced matrix protein synthesis in podocytes. *Journal of Biological Chemistry*.

[53] Bennet W. M., Connacher A. A., Scrimgeour C. M., Jung R. T., Rennie M. J. (1990). Euglycemic hyperinsulinemia augments amino acid uptake by human leg tissues during hyperaminoacidemia. *American Journal of Physiology-Endocrinology and Metabolism*.

[54] Biolo G., Declan Fleming R. Y., Wolfe R. R. (1995). Physiologic hyperinsulinemia stimulates protein synthesis and enhances transport of selected amino acids in human skeletal muscle. *The Journal of Clinical Investigation*.

[55] Ferrando A. A., Chinkes D. L., Wolf S. E., Matin S., Herndon D. N., Wolfe R. R. (1999). A submaximal dose of insulin promotes net skeletal muscle protein synthesis in patients with severe burns. *Annals of Surgery*.

[56] Tesseraud S., Abbas M., Duchene S., Bigot K., Vaudin P., Dupont J. (2006). Mechanisms involved in the nutritional regulation of mRNA translation: features of the avian model. *Nutrition Research Reviews*.

[57] Huang C. Y., Yao W. F., Wu W. G., Lu Y. L., Wan H., Wang W. (2013). Endogenous CSE/H_2_S system mediates TNF-*α*-induced insulin resistance in 3T3-L1 adipocytes. *Cell Biochemistry and Function*.

[58] Manna P., Jain S. K. (2012). Vitamin D up-regulates glucose transporter 4 (GLUT4) translocation and glucose utilization mediated by cystathionine-*γ*-lyase (CSE) activation and H_2_S formation in 3T3L1 adipocytes. *Journal of Biological Chemistry*.

[59] Zhang L., Yang G., Untereiner A., Ju Y., Wu L., Wang R. (2013). Hydrogen sulfide impairs glucose utilization and increases gluconeogenesis in hepatocytes. *Endocrinology*.

[60] Mustafa A. K., Gadalla M. M., Sen N. (2009). H_2_S signals through protein S-sulfhydration. *Science Signaling*.

[61] Zhao K., Ju Y., Li S., Altaany Z., Wang R., Yang G. (2014). S-sulfhydration of MEK1 leads to PARP-1 activation and DNA damage repair. *EMBO Reports*.

[62] Brand A., Leibfritz D., Hamprecht B., Dringen R. (1998). Metabolism of cysteine in astroglial cells: synthesis of hypotaurine and taurine. *Journal of Neurochemistry*.

[63] Kaneko Y., Kimura Y., Kimura H., Niki I. (2006). L-cysteine inhibits insulin release from the pancreatic beta-cell: possible involvement of metabolic production of hydrogen sulfide, a novel gasotransmitter. *Diabetes*.

[64] Hendry W. J., Hakkak R., Cornett L. E. (1992). Selective loss of glucocorticoid-dependent responses in a variant of the DDT1MF-2 tumor cell line. *Cancer Research*.

[65] Kaneko Y., Kimura T., Taniguchi S. (2009). Glucose-induced production of hydrogen sulfide may protect the pancreatic beta-cells from apoptotic cell death by high glucose. *FEBS Letters*.

[66] Sato K., Aoki M., Kondo R., Matsushita K., Akiba Y., Kamada T. (2012). Administration of insulin to newly hatched chicks improves growth performance via impairment of MyoD gene expression and enhancement of cell proliferation in chicken myoblasts. *General and Comparative Endocrinology*.

[67] Schmidt E. K., Clavarino G., Ceppi M., Pierre P. (2009). SUnSET, a nonradioactive method to monitor protein synthesis. *Nature Methods*.

[68] Goodman C. A., Mabrey D. M., Frey J. W. (2011). Novel insights into the regulation of skeletal muscle protein synthesis as revealed by a new nonradioactive in vivo technique. *The FASEB Journal*.

