# Supplementary figures and images for: Endogenous CSE/Hydrogen Sulfide System Regulates the Effects of Glucocorticoids and Insulin on Muscle Protein Synthesis
Source: Oxid Med Cell Longev. 2019 Apr 7;2019:9752698. doi: 10.1155/2019/9752698 (PMC6476024; doi:10.1155/2019/9752698)

S2

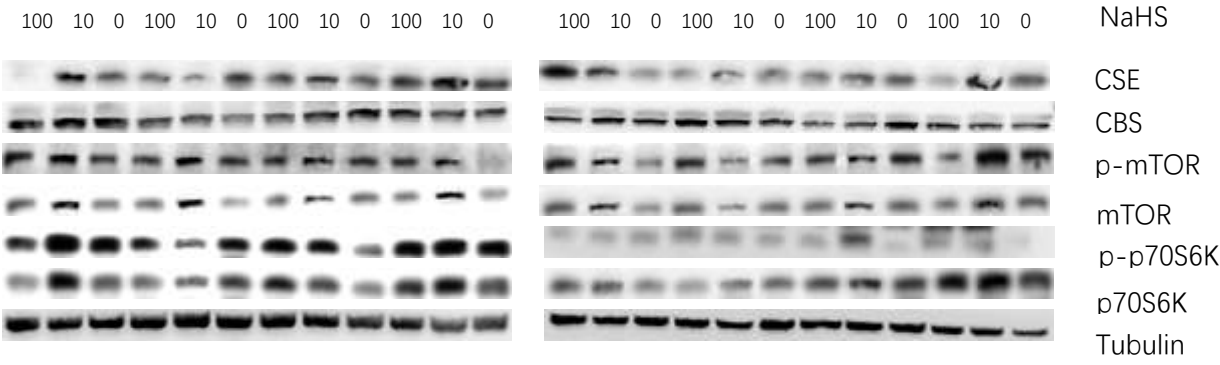

S3

A

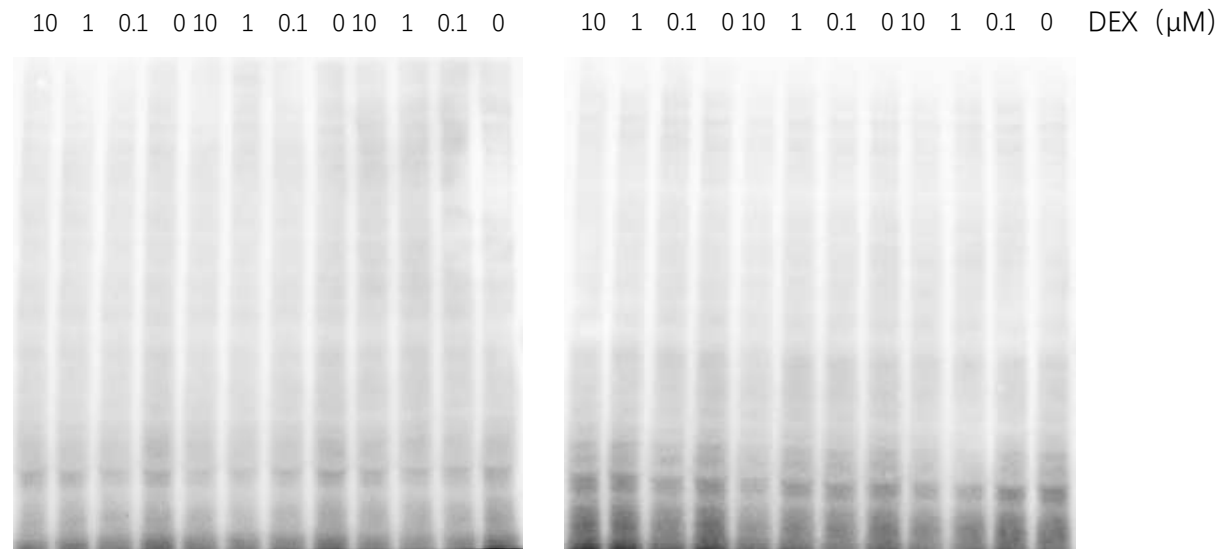

B

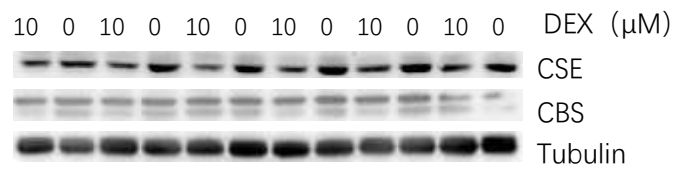

C

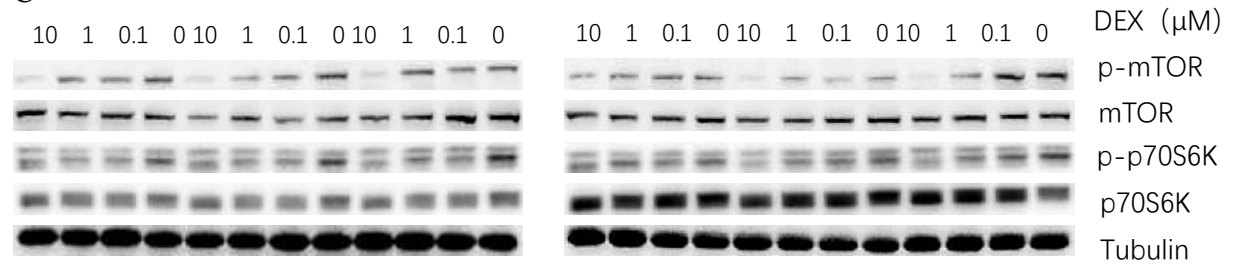

S4

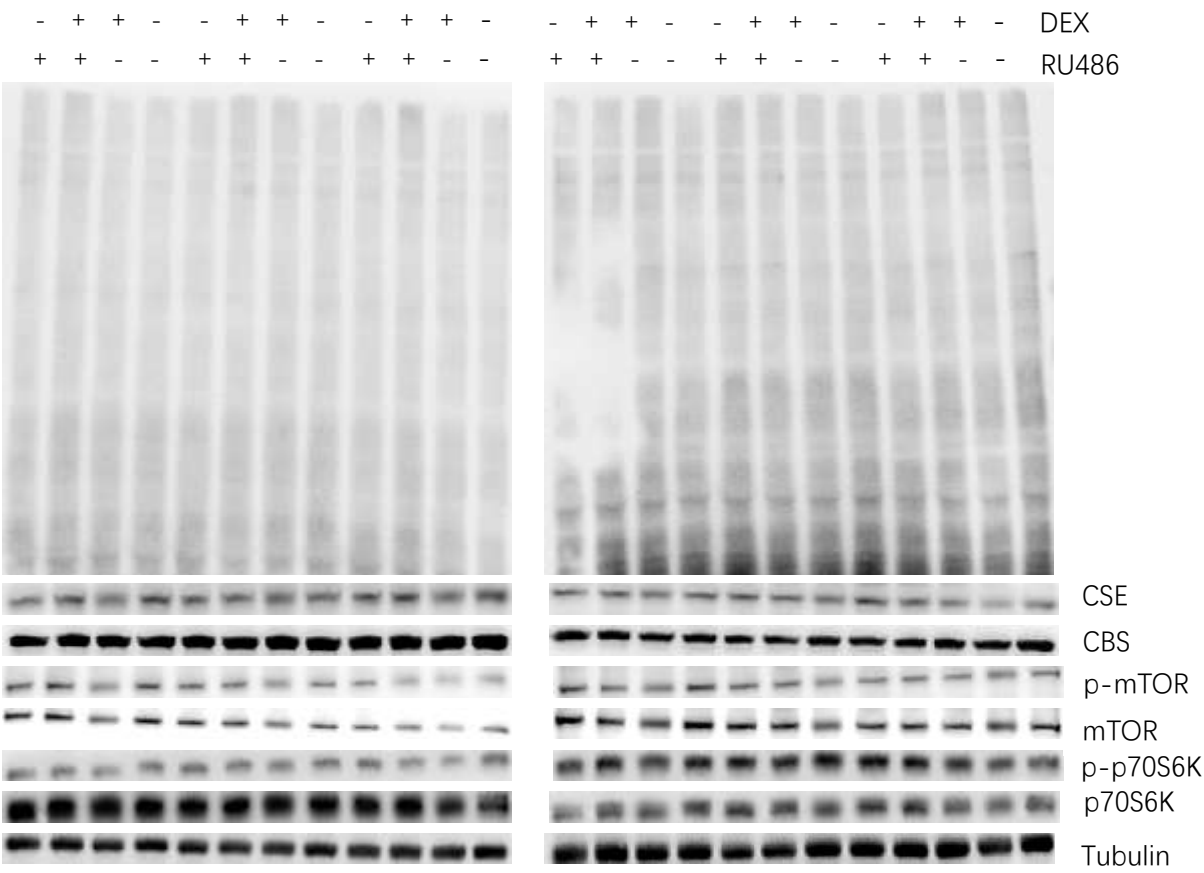

S5

A

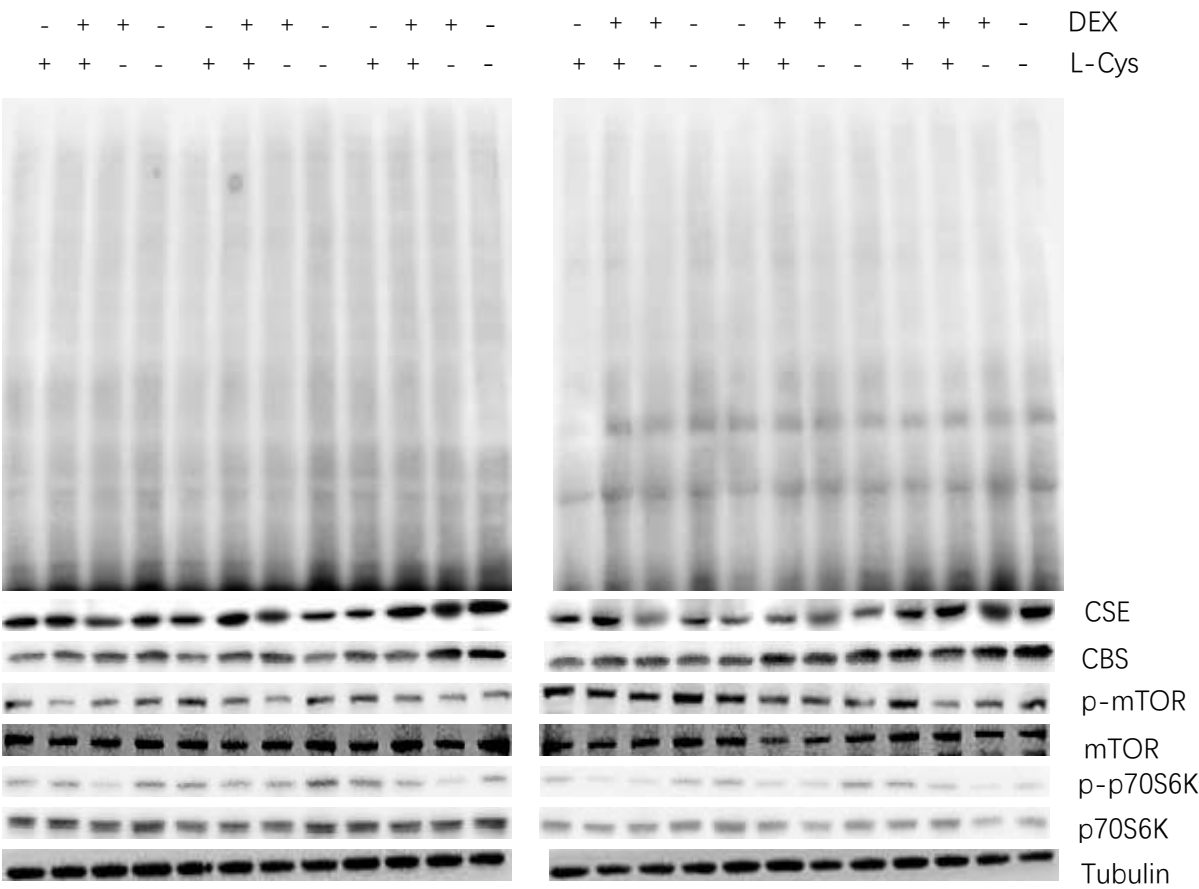

B

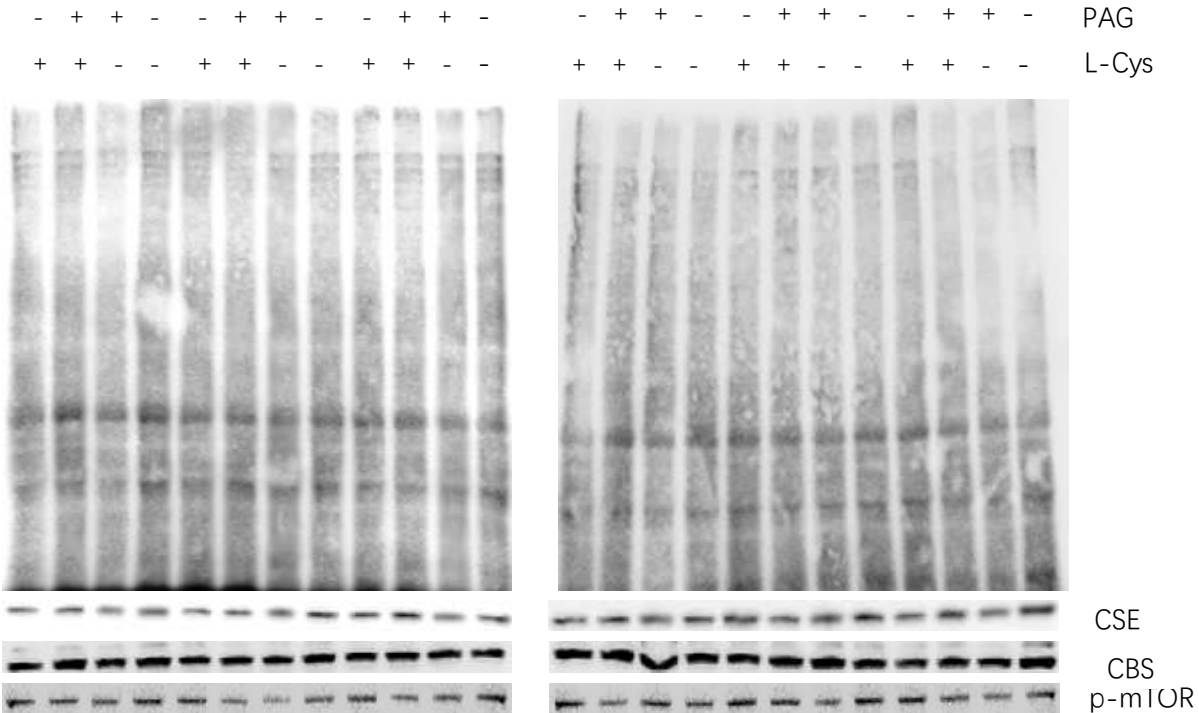

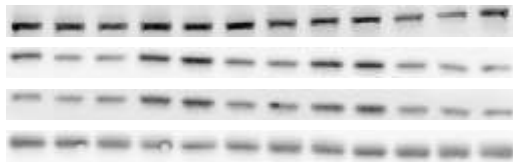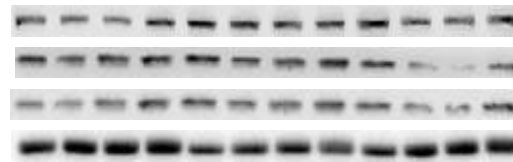

mTOR  
p-p70S6K  
p70S6K  
Tubulin

S6

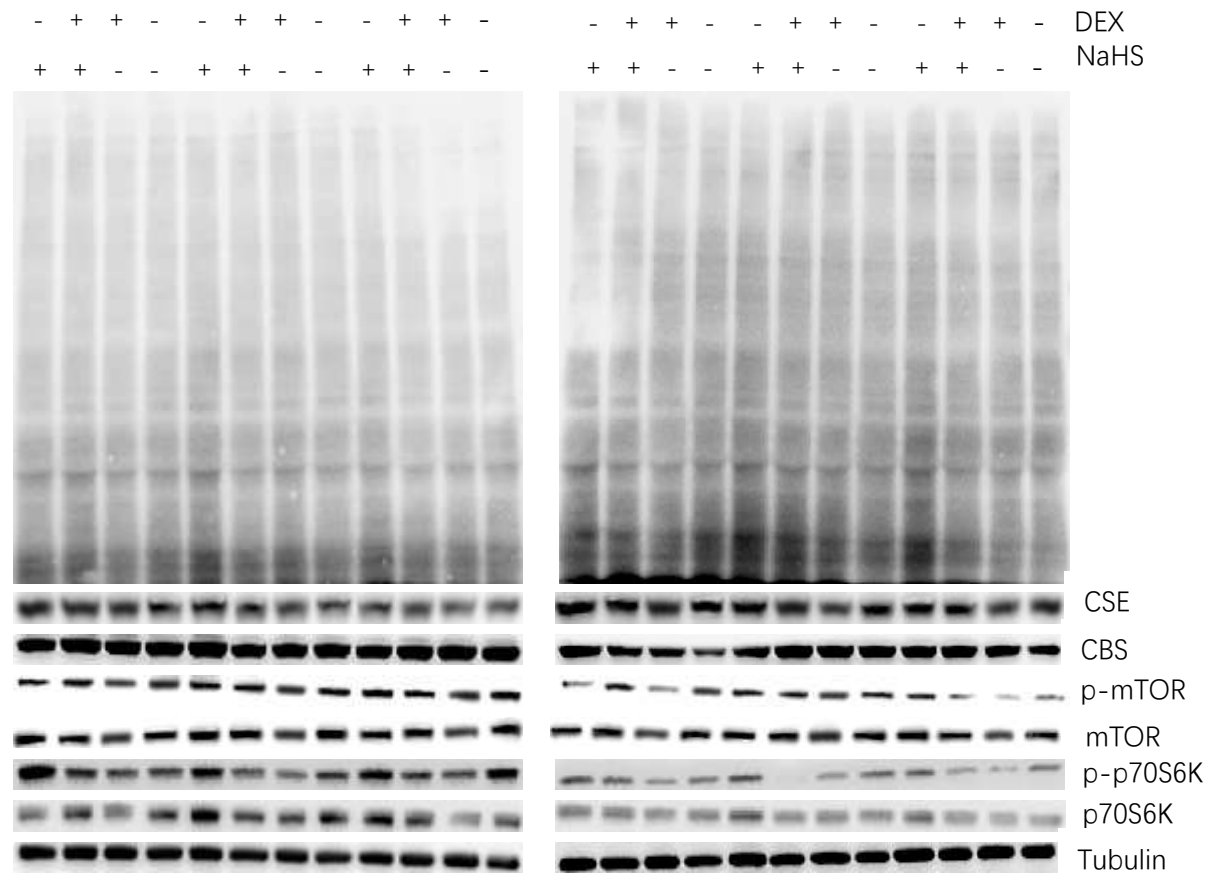

S7

|   |   |   |   |   |   |   |   |   |   |   |   |   |   |
|---|---|---|---|---|---|---|---|---|---|---|---|---|---|
| + | + | + | - | - | + | + | + | - | - | + | + | + | - |
| + | + | - | - | + | + | + | - | - | + | + | + | - | - |
| + | - | - | - | + | + | - | - | - | + | + | - | - | - |

|   |   |   |   |   |   |   |   |   |   |   |   |   |   |       |
|---|---|---|---|---|---|---|---|---|---|---|---|---|---|-------|
| + | + | + | - | - | + | + | + | - | - | + | + | + | - | DEX   |
| + | + | - | - | + | + | + | - | - | + | + | + | - | - | RU486 |
| + | - | - | - | + | + | - | - | - | + | + | - | - | - | PAG   |

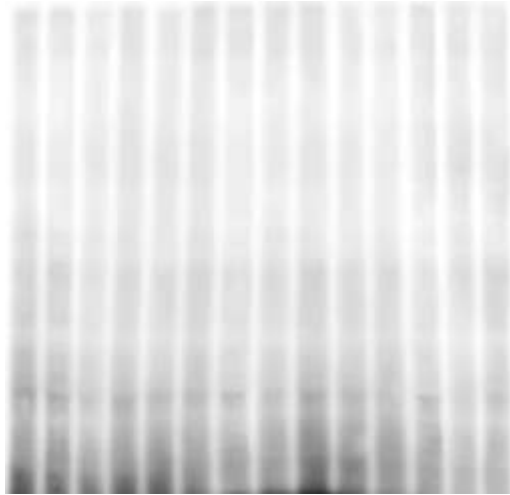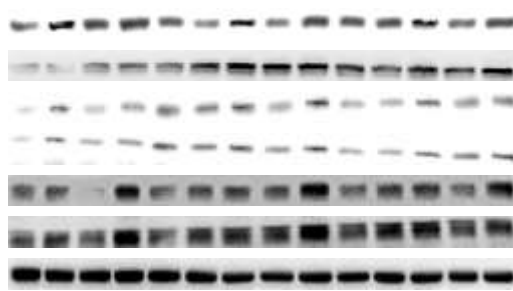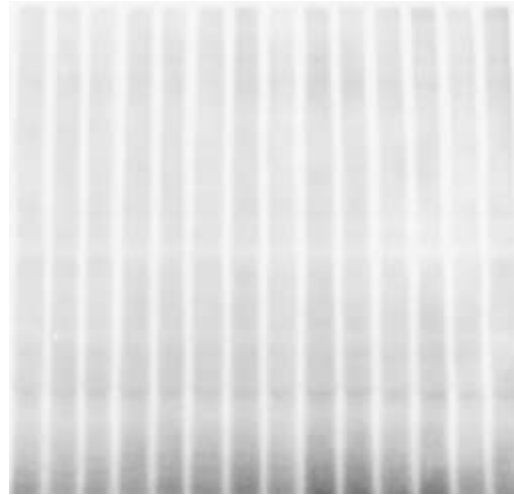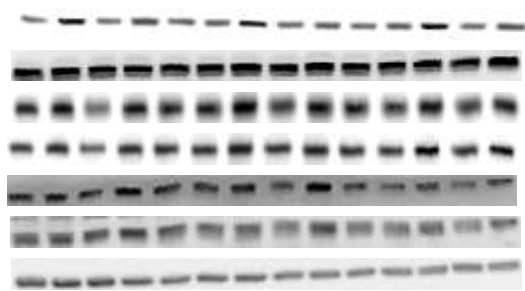

CSE  
CBS  
p-mTOR  
mTOR  
p-p70S6K  
p70S6K  
Tubulin

S8

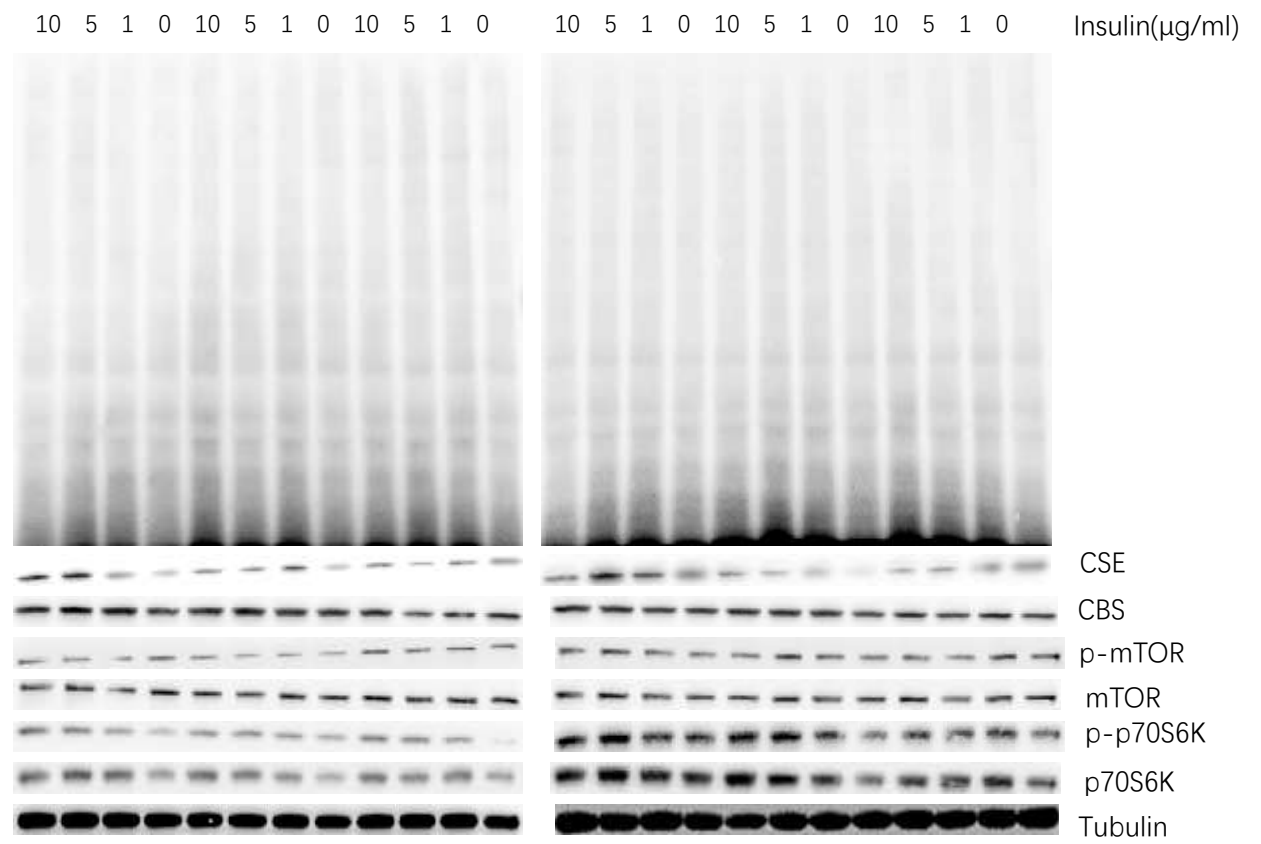

S9

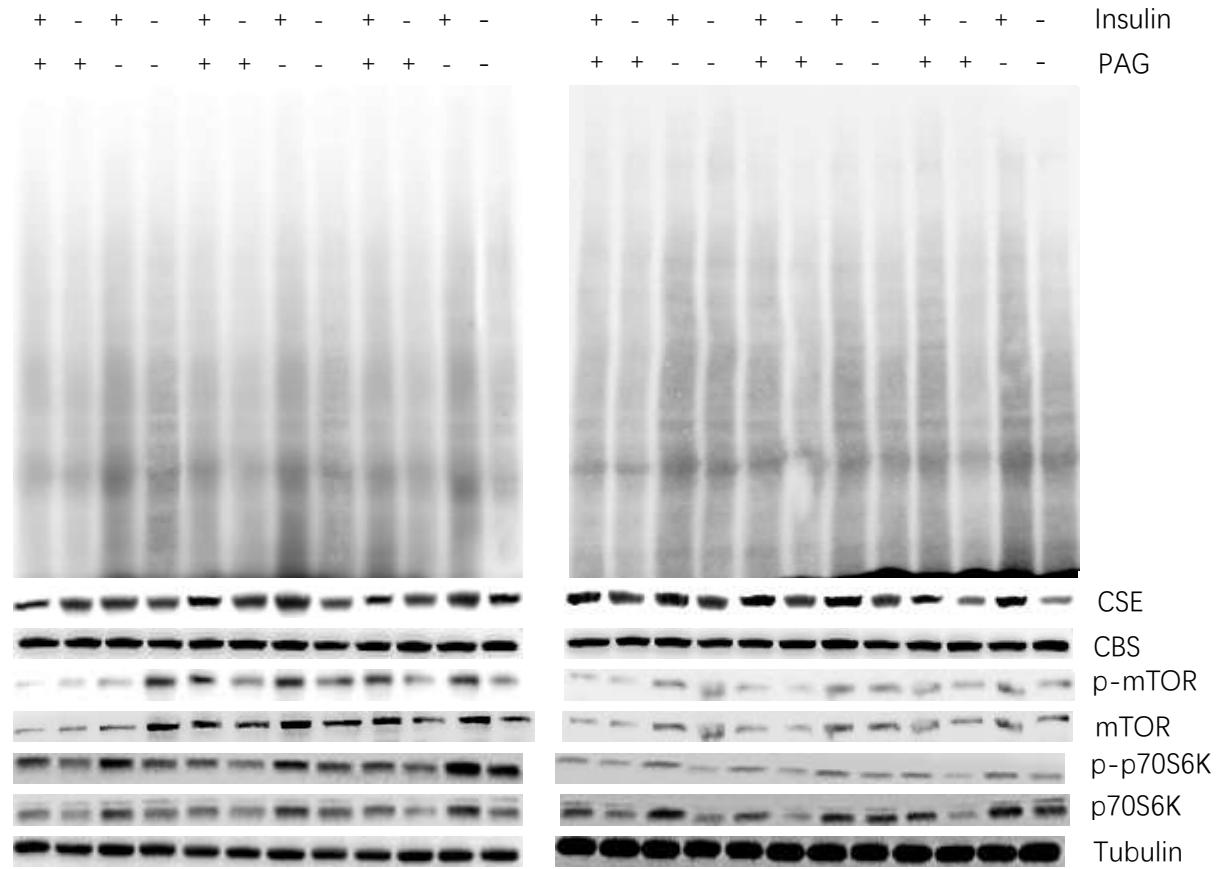

Supplement: Supplementary Materials — The relative expression of cystathionine γ-lyase (CSE), cystathionine β-synthase (CBS), and 3-mercaptopyruvate sulfur transferase (3-MST) in myoblasts of chicken ∗ P < 0.05. [file 9752698.f1.zip › Figs. S2-S9.pdf]
