# Supplementary material for: Endogenous CSE/Hydrogen Sulfide System Regulates the Effects of Glucocorticoids and Insulin on Muscle Protein Synthesis
Source: Oxid Med Cell Longev. 2019 Apr 7;2019:9752698. doi: 10.1155/2019/9752698 (PMC6476024; doi:10.1155/2019/9752698)
Supplement: Supplementary Materials — The relative expression of cystathionine γ-lyase (CSE), cystathionine β-synthase (CBS), and 3-mercaptopyruvate sulfur transferase (3-MST) in myoblasts of chicken ∗ P < 0.05. [file 9752698.f1.zip › Fig. S1.doc]

**Supplemental Fig**

The relative expression of cystathionine γ-lyase (CSE), cystathionine β-synthase (CBS), and 3-Mercaptopyruvate sulfur transferase (3-MST) in myoblasts of chicken

* P < 0.05
